# Ocular and extra-ocular features of patients with Leber congenital amaurosis and mutations in *CEP290*

**Published:** 2012-02-10

**Authors:** Suzanne Yzer, Anneke I. den Hollander, Irma Lopez, Jan-Willem R. Pott, Jan Tjeerd H.N. de Faber, Frans P.M. Cremers, Robert K. Koenekoop, L. Ingeborgh van den Born

**Affiliations:** 1 The Rotterdam Eye Hospital, Rotterdam, The Netherlands; 2 Department of Ophthalmology, Radboud University Nijmegen Medical Centre, Nijmegen, The Netherlands; 3 McGill Ocular Genetics Laboratory, Montreal Children's Hospital Research Institute, McGill University Health Centre, Montreal, QC, Canada; 4 Department of Ophthalmology, University Medical Centre Groningen, Groningen, The Netherlands; 5 Department of Human Genetics, Radboud University Nijmegen Medical Centre, Nijmegen, The Netherlands; 6 Nijmegen Centre for Molecular Life Sciences, Radboud University Nijmegen, Nijmegen, The Netherlands

## Abstract

**Purpose:**

This study investigated the centrosomal protein, 290-KD (*CEP290*) associated genotype and ocular and extra-ocular phenotype in 18 patients with Leber congenital amaurosis (LCA).

**Methods:**

Eighteen patients with LCA from 14 families with mutations in the *CEP290* gene were identified with sequencing or with heteroduplex analysis. Ophthalmic examinations were performed on all patients. Scans of the central nervous system were reassessed in three patients and obtained in two. Renal function was evaluated in all patients. Ultrasonography of the kidneys was performed in six patients.

**Results:**

Eight patients (from five families) carried the c.2991+1655A>G mutation homozygously. Nine solitary patients carried this variant combined with a nonsense, frameshift, or splice site mutation on the second allele. One new nonsense mutation was identified: c.1078C>T. Fourteen patients (from 12 families) had been completely blind from birth or had light perception. The best-recorded visual acuity was 20/200. Peripheral fundus changes appeared to be progressive with a relatively preserved posterior pole. Novel ophthalmic features for the *CEP290* phenotype were Coats-like exudative vasculopathy in two patients, a small chorioretinal coloboma in one patient, and well defined, small, atrophic spots at the level of the retinal pigment epithelium causing a dot-like appearance in five patients. Some *CEP290* patients exhibited systemic abnormalities. We found abnormal proprioception in two patients and mild mental retardation in one. One patient was infertile due to immobile spermatozoa. No renal abnormalities were detected.

**Conclusions:**

*CEP290*-associated LCA has a severe, progressive, and clinically identifiable phenotype. Distinct extra-ocular findings were noted, which may be attributed to ciliary dysfunction.

## Introduction

Leber congenital amaurosis (LCA) represents a group of clinically and genetically heterogeneous retinal dystrophies defined by severe visual dysfunction at birth, sensory nystagmus, a nondetectable electroretinogram (ERG), and an autosomal recessive inheritance pattern [1,2]. LCA has an estimated incidence of 1 in 30,000 newborns and a worldwide prevalence of approximately 200,000 patients [3,4].

Eighteen causative genes for LCA are known (see B-diseases), and mutations in these genes account for approximately 70% of all patients with LCA [5,6]. To date, the most frequently mutated LCA gene is centrosomal protein, 290-KD (*CEP290*) [7,8], which encodes a protein ubiquitously expressed in ciliated cells, including rods and cones [9]. In addition to LCA, mutations in *CEP290* can also lead to Joubert syndrome [10,11], Senior-Loken syndrome [10], the lethal disorder Meckel-Gruber syndrome [12,13], and Bardet-Biedl syndrome [14]. These diseases are termed ciliopathies, which form a group of overlapping phenotypes featuring developmental malformation of the brain, cystic kidney disease or nephronophthisis, and retinal degeneration.

*CEP290* mutations account for 6%–22% of non-syndromic LCA, depending on the population studied [5,7]. The majority of patients with LCA with *CEP290* mutations carry the intronic mutation c.2991+1655A>G (p.Cys998X), which with lymphoblast RNA analysis was found to introduce a cryptic exon into the *CEP290* mRNA, creating a stop codon at the 5′ end of the insertion. However, only approximately 50% of the mRNA contained the cryptic exon, rendering this variant a hypomorphic mutation, which subsequently may influence the phenotypic outcome [7].

In the initial report on *CEP290*-associated LCA, we reported on intrafamilial variability with respect to visual acuity in four Canadian patients, and the absence of extra-ocular symptoms, except for seizures in two affected siblings and abnormal proprioception in one patient [7]. Several reports on the *CEP290-*associated phenotype have since been published. Perrault et al. [8] described the phenotype as LCA type 1 (cone-rod), characterized by some photo-aversion, high hyperopia, severely reduced visual acuity (<20/400), and a salt-and-pepper aspect of the fundus with macular degeneration, resulting in a typical retinitis pigmentosa (RP) appearance by the second decade. Coppieters and coworkers also observed the *CEP290* LCA phenotype as one of a severe cone-rod dystrophy with progressive peripheral fundus changes, but with a relatively spared macular region [15]. Pasadhika et al. [16] studied the morphology of *CEP290*-related LCA with spectral domain–optical coherence tomography (SD-OCT). They showed preservation of the outer nuclear layer (ONL) with a poorly defined photoreceptor inner/outer segment junction and with a distorted inner retina in the central macular area. The ocular phenotype was further expanded by Littink and colleagues, who reported on two patients with *CEP290* mutations with relatively well preserved visual acuity (VA) and with detectable scotopic responses on ERG, leading the authors to diagnose these patients with early-onset severe retinal dystrophy rather than LCA [17].

Several groups studied associated extra-ocular signs in patients with LCA with *CEP290* mutations. Perrault et al. [8] observed transitory hypotonia, ataxia, mental retardation, and autistic behavior in a minority of their patients with surprising intra-familiar discrepancies. McEwen and coworkers [9] examined other ciliary-driven sensory dysfunctions, and discovered olfactory defects in retinal degeneration 16 (*rd16*) mice, and subsequently in patients with LCA with *CEP290* mutations [18]. Coppieters et al. [15] reported on different degrees of kidney disease and neurologic involvement in patients carrying the same *CEP290* mutations. The researchers hypothesized that the neurologic phenotype was modified by the presence of heterozygous Abelson Helper Integration sit 1 (*AHI1*) mutations. Papon and colleagues [19] showed ultrastructural defects in respiratory cilia in nasal biopsies and brushing on seven patients with LCA with *CEP290* mutations.

The goal of this study was to further delineate the ocular phenotype of *CEP290* LCA and to search for other extra-ocular symptoms in a cohort of Dutch and Canadian patients. In the current study of 18 patients, we were able to expand the ophthalmic phenotype of *CEP290*-related LCA by Coats-like exudative vasculopathy and chorioretinal coloboma as new ophthalmic findings.

## Methods

### Mutation analysis

DNA was extracted from peripheral blood leukocytes using a salting-out procedure [20]. Patients were tested for the c.2991+1655A>G *CEP290* mutation with allele-specific PCR [7] or with the newest version of the LCA mutation chip (Asper Ophthalmics, Tartu, Estonia). After one heterozygous allele was identified, all 53 *CEP290* coding exons were analyzed with bi-directional sequencing or heteroduplex analysis (ABI3730 and ABI3100 Genetic Analyzers; Applied Biosystems, Inc. [ABI], Foster City, CA) to identify the second allele. Primer sequences and PCR conditions were described previously (Appendix 1) [13]. Briefly, PCR amplification consisted of a denaturizing step at 95 °C for 5 min, followed by 35 cycles of amplification (95 °C for 30 s, X °C for 30 s, 72 °C for 45 s) and a final extension at 72 °C for 5 min. In two unrelated affected siblings, no blood samples were drawn at request of the parents; however, samples were available for the probands of these families.

### Patients

Eighteen healthy patients were recruited at The Rotterdam Eye Hospital and the Montreal Children’s Hospital. Written informed consent was obtained from the patients with LCA or their legal representatives before blood was drawn. The procedures were approved by the Ethics Committees of the Rotterdam Eye Hospital and McGill University Health Centre and adhered to the Declaration of Helsinki. Ten females and 8 males were included in this study in an age range from 2 to 43 years. Eight patients of six families (Patients 1, 2, 3, 4, 5, 6, 12, and 14) were part of a cohort of patients with LCA whose molecular data, but not clinical data, were described by den Hollander et al. [7]. The other ten patients were suspected of carrying *CEP290* mutations based on their fundus appearance.

After mutations in *CEP290* were identified, retrospective data were studied, and all 18 patients with LCA were reexamined. A medical history was taken and included questions about the age of onset of ocular and potential extra-ocular symptoms. The history also included questions concerning pregnancy, birth, post-natal period, development, consanguinity, and family history. Ophthalmologic examinations included evaluation of the pupillary reaction and nystagmus, best-corrected visual acuity (BCVA), and objective refractive error after cycloplegia. The anterior segments were examined with slit-lamp biomicroscopy, and funduscopy was performed after pupillary dilation. In all patients, fundus photography attempts were made. Optical coherence tomography (OCT) was obtained in four patients (OCT3 Stratus; Carl Zeiss Meditec, Inc., Dublin, CA, and Spectralis; Heidelberg Engineering, Heidelberg, Germany). ERGs were performed according to International Society for Clinical Electrophysiology of Vision standards [21]. Renal function was studied with blood sample testing for potassium, sodium, creatinine levels, and creatinine clearance in all patients. Renal ultrasounds were performed to evaluate kidney architecture in six patients.

Signs of Joubert syndrome (e.g., lack of normal decussation of superior cerebllar peduncular fiber tracts [molar tooth sign]) were specifically studied in five patients. In three patients, previously recorded images of the central nervous system were reevaluated (magnetic resonance imaging [MRI] for two and computed tomography [CT] for one), and in two other patients, MRIs were obtained.

## Results

### Mutation analysis

The results of the molecular analyses are summarized in Table 1. Fifteen patients were of Dutch descent and three of French-Canadian descent. Three sibships were included from three (Patients 1–3) and two affected individuals (Patients 8 and 9, 16, and 17). Eight patients (from five families) carried the c.2991+1655A>G mutation homozygously. Nine patients were compound heterozygotes with one c.2991+1655A>G mutation and a nonsense, frameshift or splice site mutation on the other allele. One new *CEP290* nonsense mutations was identified: c.1078C>T (p.Arg360X; Patient 15). Patient 12 was from a consanguineous marriage, but was compound heterozygous. Consanguinity was five generations ago, and segregation analysis showed the mother was a heterozygous carrier of the R1272X mutation and the father of the c.2991+1655A>G mutation. The genotype of Patients 1 (and his siblings 2 and 3), 4, 5, 6, 12, and 14 and the co-segregation of four of these families were described by den Hollander et al. [7] as patients 21365, 21393, 20152, 21918, 17971, and 12832, respectively.

**Table 1:** *CEP290* mutations in Leber congenital amaurosis patients.

		**Allele 1**		**Allele 2**	
**Patient number**	**Origin**	**Mutation**	**Effect**	**Mutation**	**Effect**
1$	Netherlands	c.2991+1655A>G	p.Cys998X	c.2991+1655A>G	p.Cys998X
2$	Netherlands	c.2991+1655A>G	p.Cys998X	c.2991+1655A>G	p.Cys998X
3$	Netherlands	c.2991+1655A>G	p.Cys998X	c.2991+1655A>G	p.Cys998X
4	Netherlands	c.2991+1655A>G	p.Cys998X	c.265dupA	p.Thr89AsnfsX1
5	Netherlands	c.2991+1655A>G	p.Cys998X	c.679_680delGA	p.Glu227SerfsX1
6	Netherlands	c.2991+1655A>G	p.Cys998X	c.180+1G>T	splice defect
7	Netherlands	c.2991+1655A>G	p.Cys998X	c.5668G>T	p.Gly1890X
8§	Canada	c.2991+1655A>G	p.Cys998X	c.2991+1655A>G	p.Cys998X
9§	Canada	c.2991+1655A>G	p.Cys998X	c.2991+1655A>G	p.Cys998X
10	Canada	c.2991+1655A>G	p.Cys998X	c.2991+1655A>G	p.Cys998X
11	Netherlands	c.2991+1655A>G	p.Cys998X	c.5587–1G>C	splice defect
12	Netherlands	c.2991+1655A>G	p.Cys998X	c.3814C>T	p.Arg1272X
13	Netherlands	c.2991+1655A>G	p.Cys998X	c.2991+1655A>G	p.Cys998X
14	Netherlands	c.2991+1655A>G	p.Cys998X	c.2991+1655A>G	p.Cys998X
15	Netherlands	c.5587–1G>C	splice defect	c.1078C>T	p.Arg360X
16¶	Netherlands	c.2991+1655A>G	p.Cys998X	c.3175dup	p.Ile1059fs
17¶	Netherlands	NT		NT	
18	Netherlands	c.4661_4663del	p.Glu1544del	c.1645C>T	p.Arg549X

*: Previously genotyped by den Hollander et al. [7]; $§¶: sibships; NT: not tested on request of parents.

### Clinical findings

Clinical information is summarized in Table 2 except for the description of the retinal appearance which is separately summarized in Table 3.On medical history, all patients had roving eye movements, or nystagmus with sluggishly pupillary reactions since early childhood. Eye poking was present in 17 patients and enophthalmos in seven patients (Patients 1, 2, 3, 5, 12, 13, and 17). Photophobia was not recorded as an early symptom in patients with vision. Two patients (Patients 2 and 15) developed photophobia later in life. Three patients (Patients 7, 14, and 15) had a clear history of night blindness.

**Table 2:** Clinical findings in Leber congenital amaurosis patients with CEP290 mutations.

		**Visual acuity**		**Refraction**		
**Patient (y)**	**Sex**	**RE**	**LE**	**RE**	**LE**	**Anterior segment and miscellaneous**
*1$ (19)	M	LP	LP	+9.25–1.00x60	+9.00 −1.25x194	iris translucency, enophthalmos
*2$ (18)	F	LP	LP	+9.25–1.75x72	+9.25–4.50x109	enophthalmos
*3$ (13)	M	NLP	NLP	+8.00–2.75x67	+8.50–3.50x140	enophthalmos, normal fundi at age 1y
*4 (8)	F	NLP	NLP	NA	NA	abnormal proprioception
*5 (5)	F	NLP	NLP	+12.00–1.50x30	+12.75–4.50x168	enophthalmos
*6 (43)	M	NLP	NLP	NA	NA	ectatic corneas with severe keratoconus, normal fundi at age (0.5y), dropped nuclei (43y), mildly mentally retarded, walked at age 6 y, abnormal proprioception
7 (39)	M	20/400	LP	11.25	+11.75–1.00x150	ASC cataract LE, visual acuity 20/200 (8y), immotile spermatozoa
8§ (15)	F	LP	LP	pseudophakia	pseudophakia	pseudophakic, cataract extraction at age 16y
9§ (16)	M	LP	LP	pseudophakia	pseudophakia	pseudophakic, keratoconus BE cataract extraction at age 10y
10 (25)	F	CF	CF	aphakia	aphakia	keratoconus RE
11 (34)	M	20/200	CF	+8.25–3.50x165	+7.50–4.25x35	visual acuity 20/100 (15y), iridotomy BE, hypofertility
*12 (9)	F	NLP	NLP	+9,75–4.50x37	+9.50–6.25x145	keratoconus BE, pigment anterior lens capsule, enophthalmos
13 (18)	F	HM	LP	+8.50–2.75x80	+7.50–3.00x100	enophthalmos, vitritis
*14 (45)	F	LP	LP	+3.75–0.75x26	+3.50–1.50x48	PSC cataract BE, asteroid hyalosis RE, sister LCA: LP, keratoconus, dots, mental retardation, epilepsia, psychiatric disorder, normal CT scan
15 (38)	F	LP	LP	−1.25–1.75x178	−3–1.25x162	PSC cataract RE, visual acuity 20/400 (23y), abnormal EEG, temporary use of anti-eileptic drugs, 2 sibs with Saethre Chotzen syndrome
16¶ (4)	F	NLP	NLP	+6.25	+6.00	keratoconus BE
17¶ (2)	M	NLP	NLP	+2.00	+3.00	enophthalmos
18 (17)	M	LP	LP	NA	NA	keratoconus BE

*: Previously genotyped by den Hollander et al. [7]; $§¶: sibships; ASC: anterior subcapsular; BE: both eyes; CF: counting fingers; CT: computed tomography; EEG: electroencephalography; F:female; HM: hand motion; LCA: Leber congenital amaurosis; LE: left eye; LP: light perception; M: male; NA: not accessible for examination; NLP: no light perception; PSC: posterior subcapsular; RE: right eye; RPE: retinal pigment epithelium; SE: spherical equivalent; y: age of examination in years.

**Table 3:** Retinal findings in Leber congenital amaurosis patients with *CEP290* mutations.

**Patient (y)**	**Recent funduscopy**
*1$ (19)	optic disc: pink, scleral rim, pseudopapillary edema; vessels: mildly attenuated; posterior pole: preserved RPE, mild wrinkling ILM, recognizable fovea without reflexes; (mid-) periphery: dot-like atrophy at RPE level, subtle RPE changes, bone-spicules
*2$ (18)	optic disc: pink; vessels: moderately attenuated; posterior pole: preserved RPE, recognizable fovea, no reflexes; (mid-) periphery: dot-like atrophy at RPE level, some bone-spicules; far periphery: more pronounced RPE atrophy
*3$ (13)	optic disc: pink, scleral rim; vessels: mildly attenuated; posterior pole: relatively hyperpigmented fovea, no reflexes, mild wrinkling ILM; (mid-) periphery: dot-like atrophy at RPE level, subtle RPE changes, sporadic bone-spicules
*4 (8)	optic disc: pink; vessels: mildly attenuated; posterior pole: relatively spared RPE, mild epiretinal membrane, recognizable fovea, no reflexes; (mid-) periphery: abnormal reflex along vascular arcade, subtle RPE changes, granular aspect
*5 (5)	optic disc: moderately pale; vessels: moderately attenuated; posterior pole: preserved RPE, recognizable fovea, no reflexes; (mid-) periphery: preserved RPE; far periphery: relatively hypopigmented, granular aspect
*6 (43)	NA
7 (39)	optic disc: pale, pseudo-papillary edema, vascular sheeting; vessels: moderately attenuated; posterior pole: preserved RPE, subtle RPE changes RE, exudative detachment LE; (mid-) periphery: pronounced RPE atrophy, bone-spicules
8§ (15)	optic disc: pink; vessels: very attenuated; posterior pole: normal; periphery: granular aspect
9§ (16)	optic disc: pink; vessels: attenuated; posterior pole: normal; periphery: normal
10 (25)	optic disc: pink; vessels: attenuated; posterior pole: recognizable fovea, diffuse hypopigmentation; (mid-) periphery: dot-like atrophy at RPE level; far periphery: pigment mottling, bone-spicules
11 (34)	optic disc: pink, scleral rim; vessels: mildly attenuated; posterior pole: preserved RPE macular region, recognizable fovea, no reflexes, mild epiretinal membrane RE; (mid-) periphery: (lobular) atrophy RPE, some bone-spicules
*12 (9)	optic disc: pink; vessels: mildly attenuated; posterior pole: preserved RPE, relatively dark, recognizable fovea, no reflexes, wrinkling ILM (BE), yellow deposits temporal to macula RE; (mid-) periphery: relatively normal
13 (18)	optic disc: pink, scleral rim; vessels: tortuous and dilated; posterior pole: relatively normal, no reflexes; (mid-) periphery: RPE atrophy, exudative retinal detachment inferior quadrants with Coat's-like vasculopathy
*14 (45)	optic disc: moderate pallor; vessels: severely attenuated; posterior pole: preserved RPE, fovea recognizable, no reflexes; (mid-) periphery: (lobular) RPE atrophy, bone-spicules
15 (38)	optic disc: mild pallor; vessels: moderately attenuated; posterior pole: preserved RPE, subtle RPE changes, ERM; (mid-) periphery: chorioretinal coloboma RE, (lobular) atrophy RPE, bone-spicules, paving-stone degeneration 360 degrees
16¶ (4)	optic disc: pink, scleral rim; vessels: mildly attenuated; posterior pole: preserved RPE, macula: relatively dark aspect; (mid-) periphery: tapetal reflex, subtle hypo-and hyperpigmented areas
17¶ (2)	optic disc: pink; vessels: mildly attenuated; posterior pole: preserved RPE; (mid-) periphery: tapetal reflex, granular aspect
18 (17)	optic disc: pink, scleral rim; vessels: moderately attenuated; posterior pole: preserved RPE, subtle RPE changes macula, present foveolar reflex RE, absent LE; (mid-) periphery: RPE atrophy, bone-spicules

*: Previously genotyped by den Hollander et al. [7]. $§¶: sibships; ERM: epiretinal membrane; ILM: internal limiting membrane; LE: left eye; NA: not accessible for examination; RE: right eye; RPE: retinal pigment epithelium; y: age of examination in years.

**Figure 1:**
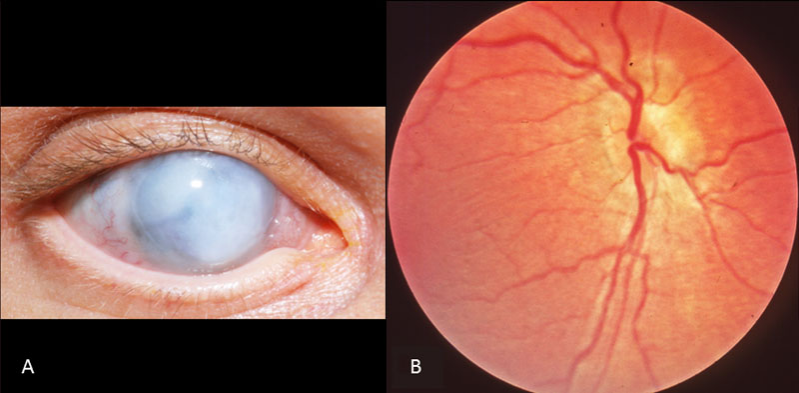
Ophthalmic features of Patient 6. **A**: External appearance of the right eye at age 43. Note the ectatic white cornea. **B**: Fundus photograph of the right eye of the same patient at the age of six months. Note the normal fundus features.

In seven patients, no light perception was recorded. In two, there was an indication of light perception in the past (Patients 4 and 6). Sixteen eyes of nine patients (Patients 1, 2, 7, 8, 9, 13, 14, 15, and 18) perceived light only. In the other patients, visual acuity varied from detecting hand motion (one eye of Patient 13) to 20/200 (one eye of Patient 11). Deterioration of visual acuity was documented in three patients over periods of 15 years or more (Patients 7, 11, and 15). Mild to high hyperopia (ranging from +2.00 to +12.00 diopters spherical equivalent) was a constant feature in the majority of the patients (11 patients). One patient showed myopia (Patient 15), possibly due to her cataract. Six patients (Patients 6, 9, 10, 12, 16, and 18) had keratoconus. In five, this was a bilateral finding. In one patient (Patient 6), ophthalmic examination was not possible due to severe ectatic corneas with vascular in-growth and bullous changes (Figure 1A). His lenses were luxated into the vitreous cavity (as seen on ultrasonography). Posterior subcapsular cataracts were observed in two patients (Patients 14 and 15) and an anterior subcapsular cataract in one (Patient 7). Two patients were pseudophakic (Patient 8 and 9), and one aphakic (Patient 10).

**Figure 2:**
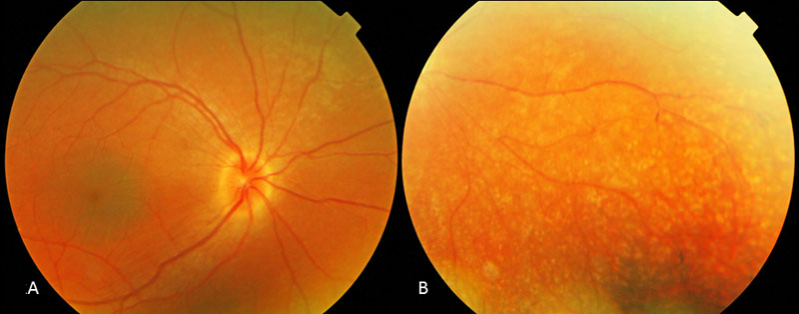
Fundus photographs of the right eye of Patient 3 at age 13. **A**: Note the pink optic disc with scleral rim, mild attenuation of vessels, and preservation of the RPE in the posterior pole, with greyish, darker appearance of the macula with subtle wrinkling of the inner limiting membrane. **B**: Note the subtle, atrophic, well defined dot-like changes at the level of the RPE and the bone spicules in the periphery.

**Figure 3:**
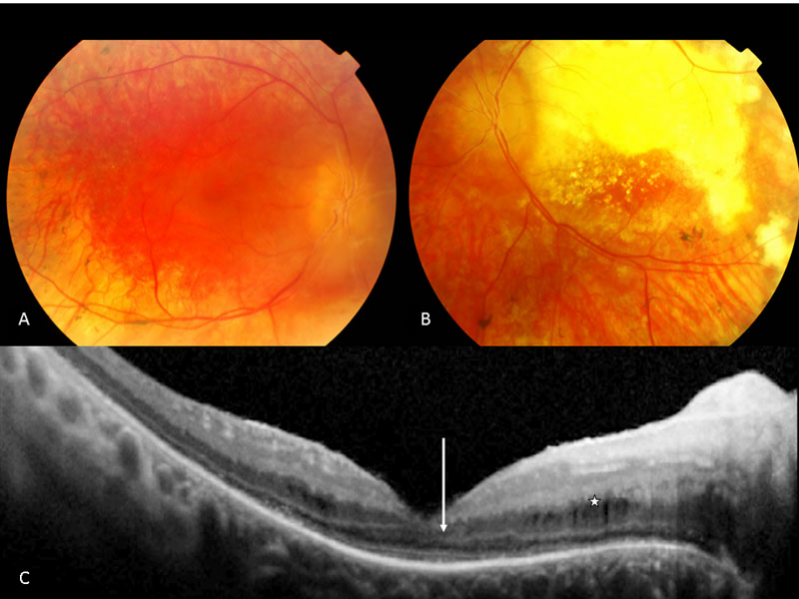
Fundus photographs and OCT of Patient 7 at age 39. **A**: Note the pseudopapillary edema, vascular sheathing, preserved RPE in the posterior pole, subtle RPE changes in the macula, and the pronounced RPE and choriocapillary atrophy with bone spicule-like pigmentations along the vascular arcade in the right eye. **B**: Note the exudation in the posterior pole with shallow detachment in the left eye. **C**: Spectralis optical coherence tomography (OCT) image of the macular region of the right eye showing cyst-like changes (*) in the outer nuclear layer with preservation of the inner/outer segment junction under the fovea but absent in the parafoveal region. Note the thinning of the outer nuclear layer at the center (arrow).

**Figure 4:**
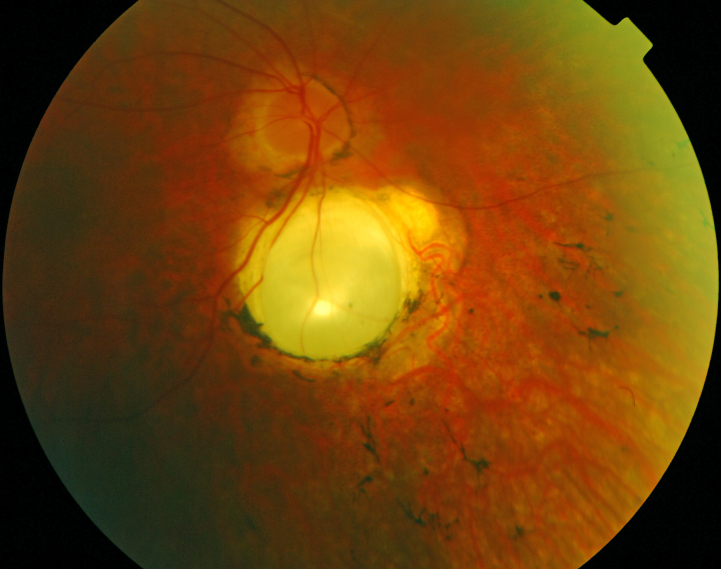
Fundus photograph of the right eye of Patient 15 at age 38. Note the mild pallor of the optic disc, the attenuation of the vessels, the chorioretinal coloboma, the retinal pigment epithelium atrophy (RPE) atrophy, and the bone spicule-like pigmentations.

Seventeen patients were available for funduscopy. A relatively pink optic disc was observed in 13 patients (Patients 1, 2, 3, 4, 8, 9, 10, 11, 12, 13, 16, 17, and 18; Figure 1B and Figure 2A). In six (Patients 1, 3, 11, 13, 16, and 18), a distinct yellowish scleral rim was observed (Figure 2A). Two patients (Patients 1 and 7) had pseudo-papillary edema with epipapillary fibrosis in one (Patient 7; Figure 3A). Another patient (Patient 15) displayed a small chorioretinal coloboma inferior to the optic disc unilaterally (Figure 4). Vessels were mildly to moderately attenuated in all but one patient whose vessels were dilated (Patient 13). One patient (Patient 7) showed vascular sheathing on the optic disc (Figure 3A).

All 17 patients had a preserved posterior pole. The macula and fovea were recognizable in all patients but without normal reflexes. The right eye in Patient 18 was the only exception because it showed a foveal reflex. The macular region was relatively darker pigmented compared to the surrounding RPE in three patients (Patients 3, 12, and 16) (Figure 2A). In four patients (Patients 7, 10, 15, and 18), the macular region displayed subtle RPE changes. In six patients (Patients 1, 3, 4, 11, 12, and 15), mild wrinkling of the inner limiting membrane with or without epiretinal fibrosis was noted.

**Figure 5:**
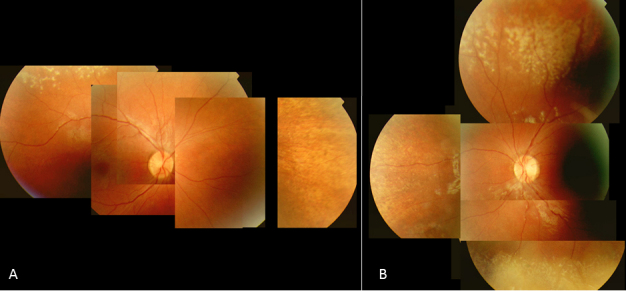
Fundus photographs of the right eye (**A**) and left eye (**B**) of Patient 16 at age 4. The optic nerve is pink with mild attenuation of the vessels and a preserved macula. Note the grayish white, patchy, tapetal reflex in the midperiphery.

Two patients did not have peripheral RPE changes (Patient 9 aged 16 and Patient 12 aged 9). In five patients, including a sibship of three affected (Patients 1, 2, 3, 10, and 13), small atrophic spots at the level of the RPE that imposed as dot-like changes were noted in the periphery combined with a granular aspect of the RPE (Figure 2B). Three of these patients (Patients 2, 3, and 13) also displayed some intra-retinal pigmentations. A striking greyish white, patchy tapetal reflex was seen in the (mid-) periphery in three young patients (Patients 4, 16, and 17; Figure 5A,B). One (Patient 16) had hypo- and hyper-pigmented areas in the far periphery whereas her younger brother (Patient 17) did not. In the remaining patients (Patients 5, 7, 8, 11, 14, 15, and 18) ranging in age from five to 39, RPE atrophy was more pronounced with bone spicule-like pigmentations.

Two patients (Patients 7 and 13) were treated for Coats-like exudative vasculopathy. Patient 7 presented with Coats-like exudative changes in the left eye at the age of 31 and developed telangiectatic vessels in the inferior quadrants of the right eye at the age of 38 (Figure 3B). The other patient (Patient 13) returned a year after her last check-up because of photopsia and was found to have exudative detachments with tortuous, dilated vessels in the inferior quadrants of both eyes at the age of 18.

**Figure 6:**
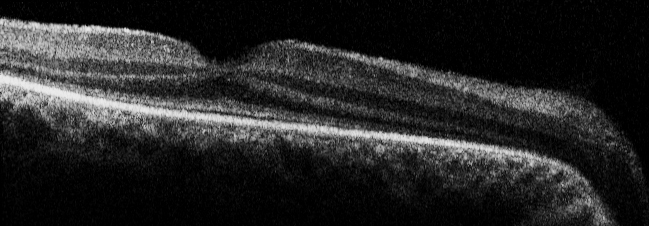
Spectralis OCT of the right eye of Patient 12 at age 9. Note the intact and normal thickness of all retinal layers.

Goldmann perimetry was performed in two patients (Patients 7 and 11) and revealed central visual fields of less than 5° using the largest target (V-4e). ERG responses were nondetectable in all patients. OCT images revealed normal-appearing retinal layers in Patient 12 (Figure 6), whereas thinned photoreceptor and outer nuclear layers were observed in two patients with deep foveal pits (Patients 7 and 14) and cyst-like changes in one of them (Patient 7; Figure 3C). These cysts were not noted on funduscopy. In another patient (Patient 11), the thickness of the retinal layers seemed normal, but foveal depression was lacking.

The motor and intellectual development in 16 patients was reported and documented as normal when compared to other blind children. None of our patients had a hearing deficit, polydactyly, or obesity. Olfactory dysfunction was not reported. Two patients (Patients 4 and 6) had abnormal proprioception, and one had mild mental retardation. No abnormalities suggestive of Joubert syndrome were present on MRI, although minor anomalies were seen in one patient with normal intelligence (Patient 4). Neuro-imaging in another four patients (Patients 1, 5, 7, and 12) did not reveal any abnormalities of the central nervous system. Electroencephalography abnormalities were recorded in the past in one patient (Patient 15) for which she received treatment with antiepileptic drugs.

All patients had normal renal function on laboratory testing. No signs of cystic kidney disease were detected on ultrasonography in the patients (Patients 1, 4, 5, 6, 7, and 12) tested.

Three male patients were of reproductive age. Two did not have offspring: one had mental retardation (Patient 6), and the other one (Patient 7) was infertile due to immobile spermatozoa. Patient 11 had hypofertility but had conceived offspring naturally.

## Discussion

The goal of the current study was to investigate ocular specific characteristics and extra-ocular features in patients with *CEP290*-related LCA.

In our cohort of 18 patients, we found one new nonsense mutation (Patient 15; c.1078C>T [p.Arg360X]) in *CEP290*. The most frequently encountered *CEP29*0 mutation in patients of European descent with LCA [7,8], the c.2991+1655A>G mutation, was detected in 22 of 34 alleles (of 14 families) tested. Almost half of the patients carried a homozygous c.2991+1655A>G mutation. One third of the patients had this mutation combined with a nonsense, frameshift, or splice site mutation on the other allele. In only two patients, the founder mutation was not detected, but the ocular phenotype of these patients was identical to the other patients.

Because of the relative large number of patients with LCA with *CEP290* mutations in the present study spread over different age groups, we were able to study different stages of the disease. Although there was some inter- and intrafamilial variability, the patients showed a relatively homogenous distinctive phenotype. We found frequently occurring symptoms as in other forms of LCA, such as eye poking, enophthalmos, nystagmus, sluggish pupillary reflexes, hyperopia, keratoconus, and juvenile cataracts [22,23]. Visual acuity in the majority of the patients was either light perception or no vision at all from birth. In the small group of patients with visual acuity equal to or more than hand motion, we were able to record progressive deterioration of visual acuity in succeeding years. Although we did not perform a longitudinal study, with retrospective data analysis (including fundus color photographs), we established an increase in degenerative changes on funduscopy, from no changes to small, well defined, atrophic spots at the level of the RPE (dot-like) to more pronounced RPE atrophy with intraretinal bone spicule-like pigmentations and a preserved macular region as in RP. Based on VA loss and progressive retinal changes over time, we conclude that LCA caused by *CEP290* mutations is a progressive retinal dystrophy.

Perrault et al. [8] and Coppieters et al. [15] classified *CEP290* associated LCA as a cone-rod retinal dystrophy. Whereas no ERG data were available in the first, the latter mentioned severe cone-rod disease on ERG testing in three out of 27 patients with LCA and *CEP290* mutations. Littink and colleagues [17] observed two patients with *CEP290* mutations, relatively well preserved VA (up to 20/40), and only detectable rod function and no cone function on ERG. Although we did not have recordable ERG responses in any of the patients studied, our clinical data of progressive peripheral retina changes and a relatively spared macula even in late stages of the disease suggest that the patients in this cohort had a progressive rod-cone type of disease rather than a cone-rod one.

We were able to get spectral domain–optical coherence tomography (SD-OCT) in four of our patients. Thinning of the ONL and the photoreceptor layer was found in two. The other patients displayed normal retinal layers at the macula (Figure 6); however, the foveal pit was absent in one. Other OCT studies in LCA *CEP290* patients also showed the presence of all retinal layers [17]. Pasadhika et al. [16] showed that the photoreceptor inner/outer segment juncture was poorly defined in the central macula whereas this junction was invisible in the periphery. A retained ONL was found in the central macular area of most patients that was more prominent at younger age. Inner retinal structures were distorted, and about half of the studied patients had cystlike lesions in the inner retina, similar to one of our patients (Figure 3C). Cideciyan et al. [24] performed a retinal micro-anatomic OCT study and revealed a relatively preserved foveal cone photoreceptor lamination and detectable subjacent RPE in patients with LCA and *CEP290* mutations, independent of severity of visual loss. The sparing of cones, however, decreased with aging, suggesting that *CEP290* mutant central retinas show slowly progressive degeneration of cones. Cideciyan et al. [24] also found that cone cell death seemed considerably slower than the loss of rods, suggesting different roles of *CEP290* in the two photoreceptor populations. These findings all further support our impression that from a morphological perspective in fact this type of retinal dystrophy might be a rod-cone disease.

As for the retinal appearance, in three patients we observed a striking tapetal reflex, consisting of intraretinal greyish white marbled areas. In the younger siblings, the tapetal reflex was more pronounced than in the older patient, suggesting the possibility of a transient phenomenon, which was also documented by Littink et al. [17]. One of these patients carried the intron mutation homozygously whereas the other was a compound heterozygote. Perrault et al. [8] depicted the same feature in one of their patients carrying the c.2991+1655A>G mutation homozygously.

The small atrophic spots at the RPE layer and the tapetal reflex–like changes seem specific to *CEP290*-associated LCA, since they have not been described in other forms of LCA. Another new observation was a distinct yellow scleral rim and pseudopapillary edema in a subset of patients. A scleral ring is a relatively common finding in RP [25] and RP-related disorders but seems suggestive of this specific type of LCA.

Two of our patients developed bilateral Coats-like exudative vasculopathy, a rare complication in patients with RP (1.2%–3.6%) and frequently associated with juvenile RP caused by Crumbs like 1 gene (*CRB1*) mutations [26]. However, Coats-like exudative vasculopathy has never been described in LCA. In a review paper, Kahn et al. [27] referred to a report on LCA and Coats-like disease. In retrospect, this report represents a family with early-onset RP. Our finding of bilateral Coats reaction in two patients (both homozygous for the c.2991+1655A>G mutation) suggests mutations in *CEP290* might predispose for this type of complication. Senior-Loken syndrome, which can also be caused by *CEP290* mutations [10], has also been shown to be associated with Coats-like disease [28].

Another new finding was the chorioretinal coloboma in one of our patients who did not carry the founder mutation. The occurrence of a developmental coloboma may be coincidental but was reported before in a Dutch patient with Joubert syndrome [29] for whom no molecular data are available.

Concerning the extra-ocular symptoms, one of our male patients (Patient 7) was infertile due to immobile spermatozoa, and one patient was hypofertile, presumably due to asthenospermia. This finding was reported previously [19] in a compound heterozygous *CEP290* LCA patient. It seems logical to assume that *CEP290* mutations may cause abnormal sperm tails and velocity. The observation that axonemes of spermatozoa were abnormal in groups of patients with X-Linked RP [30] and Usher syndrome [31] was made more than 20 years ago before the identification of ciliary genes, such as retinitis pigmentosa type 1(*RP1*), retinitis pigmentosa GTPase regulator gene (*RPGR*), retinitis pigmentosa GTPase regulator-interacting protein (*RPGRIP1*), and the Usher syndrome genes [32]. Infertility in mice with overexpression of *RPGR* has been reported recently [33]. Since other cilia-driven problems were proven, such as ultrastructural respiratory cilia defects and olfactory dysfunction in patients with *CEP290* mutations and heterozygote carriers [19], further investigation of sperm abnormalities in male patients and male heterozygotes would be interesting.

Abnormalities of the central nervous system such as the molar tooth sign in Joubert syndrome were excluded in five of our patients including two patients with balance difficulties one of whom had mild mental retardation. These two ataxic patients had the c.2991+1655A>G mutation combined with a severe mutation (frame shift or a splice site) on the other allele. Perrault and coworkers also found mental retardation and/or autistic disorders in three *CEP290-*associated LCA families with homozygous or compound heterozygous frameshift mutations. One family had the molar tooth sign, whereas neuro-imaging was not available for the other two families [8]. No abnormalities suggestive of renal disease were detected in our cohort, and no renal cysts were found in the six patients tested.

Altogether, in our study *CEP290*-LCA seemed to be associated with a predominantly ocular phenotype. Although, we did not examine all our patients in depth for other possible extra-ocular features such as anosmia and respiratory dysfunction, indicating aberrant ciliary functioning. Patients homozygous for the hypomorphic c.2991+1655A>G mutation might have more residual protein than patients with compound heterozygous mutations, and therefore might present with a less severe disease [7]. In this cohort of patients, however, visual acuity was best in patients who were compound heterozygotes, and extra-ocular features occurred in homozygous (mental retardation) and in compound heterozygous patients (ataxia, mental retardation, and infertility). Therefore, other genetic variants may modify the phenotype as described by Leitsch et al. [14], who reported on a Bardet-Biedl syndrome (BBS) patient with homozygous *CEP290* mutations and one heterozygous mutation in another ciliary BBS gene (Meckel syndrome type 3 [*MKS3*]). Recently, Coppieters and colleagues hypothesized the influence of heterozygous *AHI1* mutations on the *CEP290* LCA phenotype. Khanna et al. [34] demonstrated that a polymorphic coding variant in retinitis pigmentosa GTPase regulator-interacting protein-1 like (*RPGRIP1L*) influences the development of retinal degeneration in individuals with ciliopathies caused by mutations in other genes. Therefore, the predictive value of the genotype seems more complicated than generally assumed and probably depends on the degree of complex disruptions.

With the recent promising developments in human gene replacement therapy in LCA caused by *RPE65* mutations [35-37], treatment options for LCA caused by *CEP290* mutations may become available in the future. Since this form of LCA occurs frequently, the accompanying phenotype should be recognized. We conclude that the LCA phenotype resulting from *CEP290* mutations in a Dutch and French-Canadian cohort is a severe, slowly progressive form of LCA with a clinically identifiable ocular phenotype. The phenotype consists of a relative normal optic disc with scleral ring, mild or moderate attenuation of the vessels, a relatively preserved posterior pole, and some mild lobular RPE atrophy without obvious bone spicules in the periphery that progresses with aging. New clinical ocular findings were small, atrophic RPE spots, Coats-like exudative vasculopathy, and chorioretinal coloboma. A tapetal reflex was observed as a possible transient phenomenon. New interesting extra-ocular findings were immotile spermatozoa and ataxia. Extra-ocular findings in patients with LCA with *CEP290* mutations are relatively frequent and mutation independent.

## Appendix 1. Sequences and PCR conditions of *CEP290*.

Abbreviations: °C represents degrees celcius; bp represents base pairs; F represents forward; MgCl_2_ represents magnesium; chloride; R represents reverse. To access the data, click or select the words “Appendix 1.” This will initiate the download of a compressed (pdf) archive that contains the file.
